# Supplementary material for: Energy Balance and Risk of Mortality in Spanish Older Adults
Source: Nutrients. 2021 May 4;13(5):1545. doi: 10.3390/nu13051545 (PMC8147789; doi:10.3390/nu13051545)
Supplement: Supplementary file 1 [file nutrients-13-01545-s001.zip › nutrients-1131600-supplementary.pdf]

Table S1. Baseline characteristics of the participants with no available follow-up data compared to those with at least one follow-up data on energy intake

	**At least 2 data points**	**Only baseline**	***P*-value [a]**
N	6180	939	
Women, n (%)	3,570 (57.8)	542 (57.7)	0.98
Age, y	67.0 (6.1)	66.9 (6.7)	0.75
Energy intake baseline kcal, mean (SD)	2,242 (539)	2,193 (563)	0.01
Mediterranean diet adherence score (0-14), mean (SD)	8.71 (1.90)	8.41 (1.85)	<.0001
Physical activity METs.min/day, mean (SD)	236.8 (242.1)	192.5 (206.2)	<.0001
Alcohol intake g/day, mean (SD)	8.4 (14.2)	7.3 (12.8)	0.02
BMI kg/m^2, mean (SD)	29.9 (3.8)	30.3 (4.1)	0.01
Hypertension, n (%)	5,101 (82.5)	788 (83.9)	0.30
Hypercholesterolemia, n (%)	4,473 (72.4)	670 (71.4)	0.51
Hypertriglyceridemia, n (%)	1,780 (28.8)	255 (27.2)	0.30
Current smokers, n (%)	1,517 (24.6)	215 (22.9)	0.01
Diabetes, n (%)	2,980 (48.2)	489 (52.1)	0.03

Figure S1. Association between long-term ratio of energy intake to theoretical energy requirement and mortality risk in a restricted sample with two data points (N=6,180), PREDIMED Study

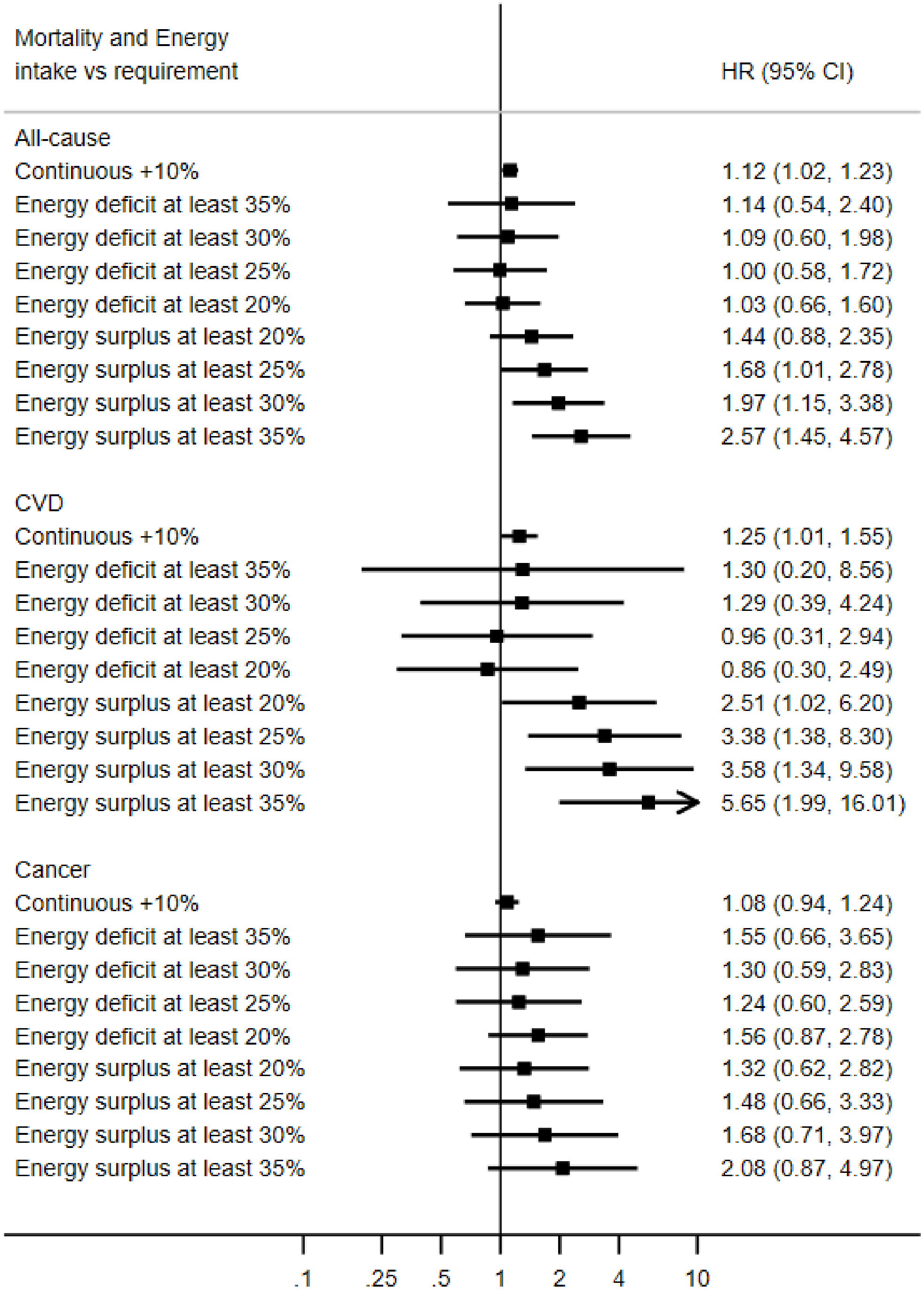

Values are multivariable hazard ratios HRs and 95% confidence intervals, stratified by sex, study centre and education level, and adjusted for baseline age, intervention group, hypertension, hypertriglyceridemia, hypercholesterolemia, diabetes, alcohol intake, smoking status and cumulative average of Mediterranean diet score.

Figure S2. Association between long-term ratio of energy intake to theoretical energy requirement and mortality risk after exclusion of “over-eaters” and “under-eaters” from the reference category, PREDIMED Study

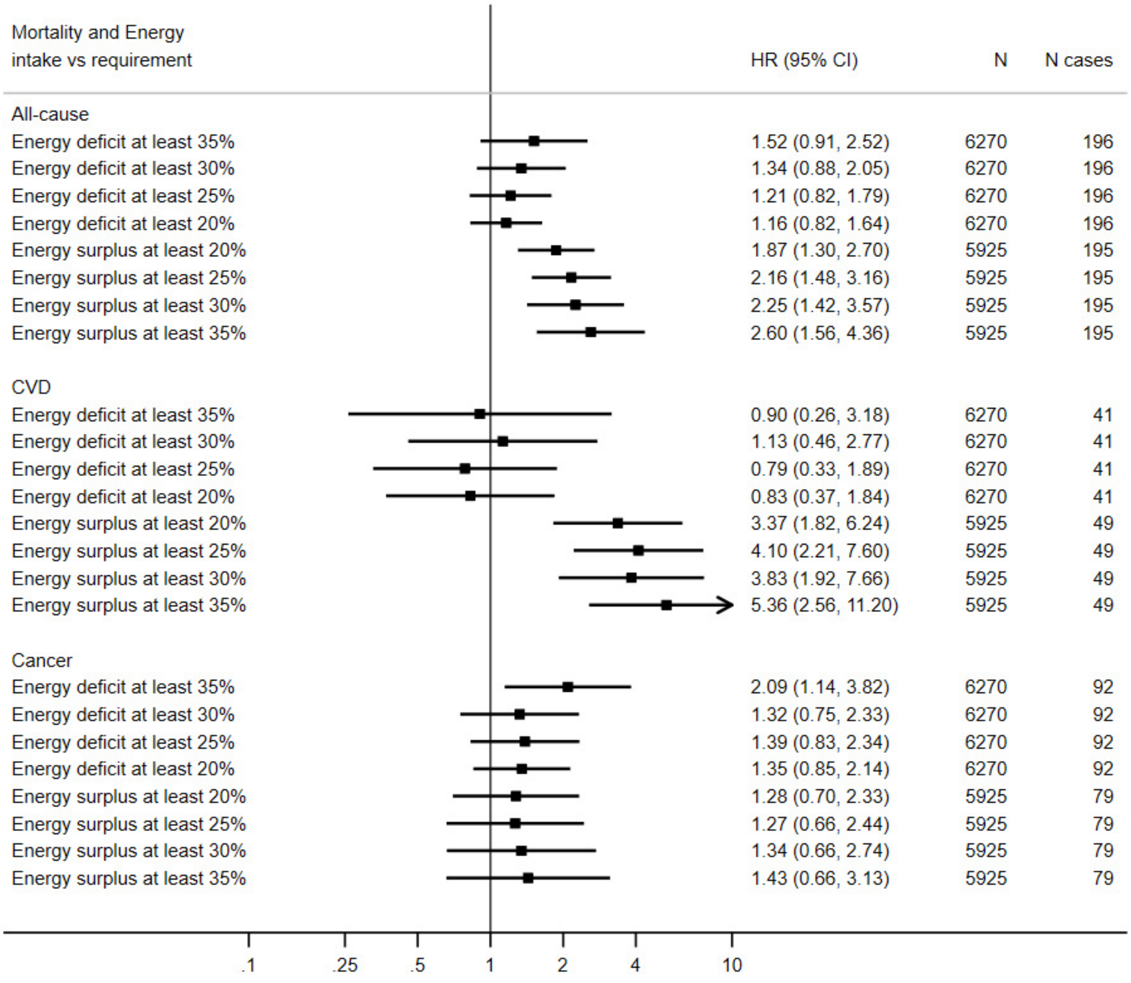

Values are multivariable hazard ratios HRs and 95% confidence intervals, stratified by sex, study centre and education level, and adjusted for baseline age, intervention group, hypertension, hypertriglyceridemia, hypercholesterolemia, diabetes, alcohol intake, smoking status and cumulative average of Mediterranean diet score. For the “calorie restriction” analyses, the reference category excluded people with ratio energy intake/requirement >25%. For the “calorie excess” analyses, the reference category excluded people with ratio energy intake/requirement <-25%.

Figure S3. Association between change in energy intake from baseline and mortality risk after exclusion of “over-eaters” and “under-eaters” from the reference category, PREDIMED Study

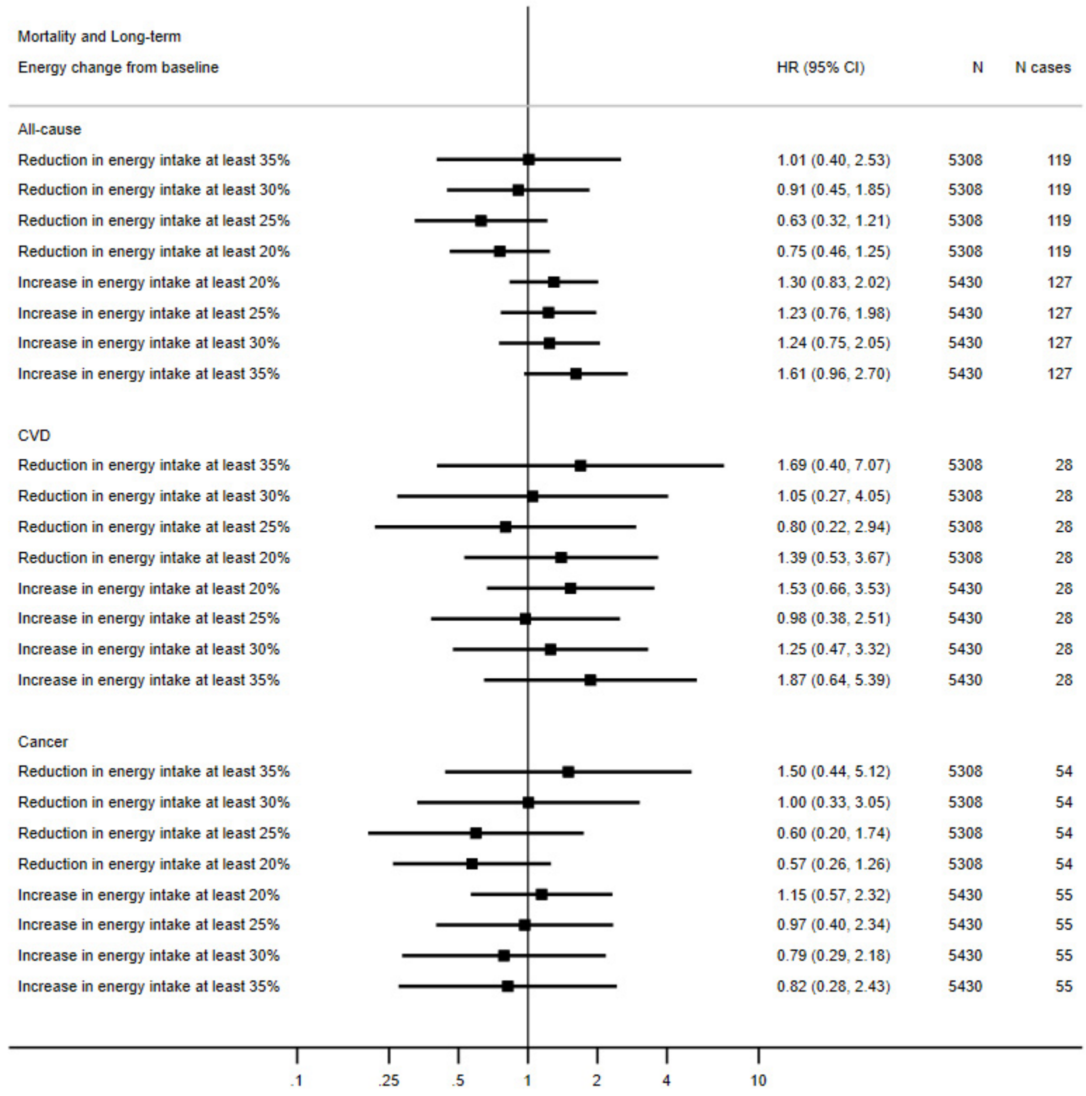

Values are multivariable hazard ratios HRs and 95% confidence intervals, stratified by sex, study centre and education level, and adjusted for baseline age, intervention group, hypertension, hypertriglyceridemia, hypercholesterolemia, diabetes, alcohol intake, smoking status and cumulative average of Mediterranean diet score. For the “calorie restriction” analyses, the reference category excluded people with ratio energy intake/requirement >25%. For the “calorie excess” analyses, the reference category excluded people with ratio energy intake/requirement <-25%.

Figure S4. Effect of adjustment for cardiovascular risk factors, baseline body mass index and change in body mass index on the estimates of associations with all-cause, cardiovascular and cancer mortality of the ratio of energy intake to energy requirement, N=6720, PREDIMED study

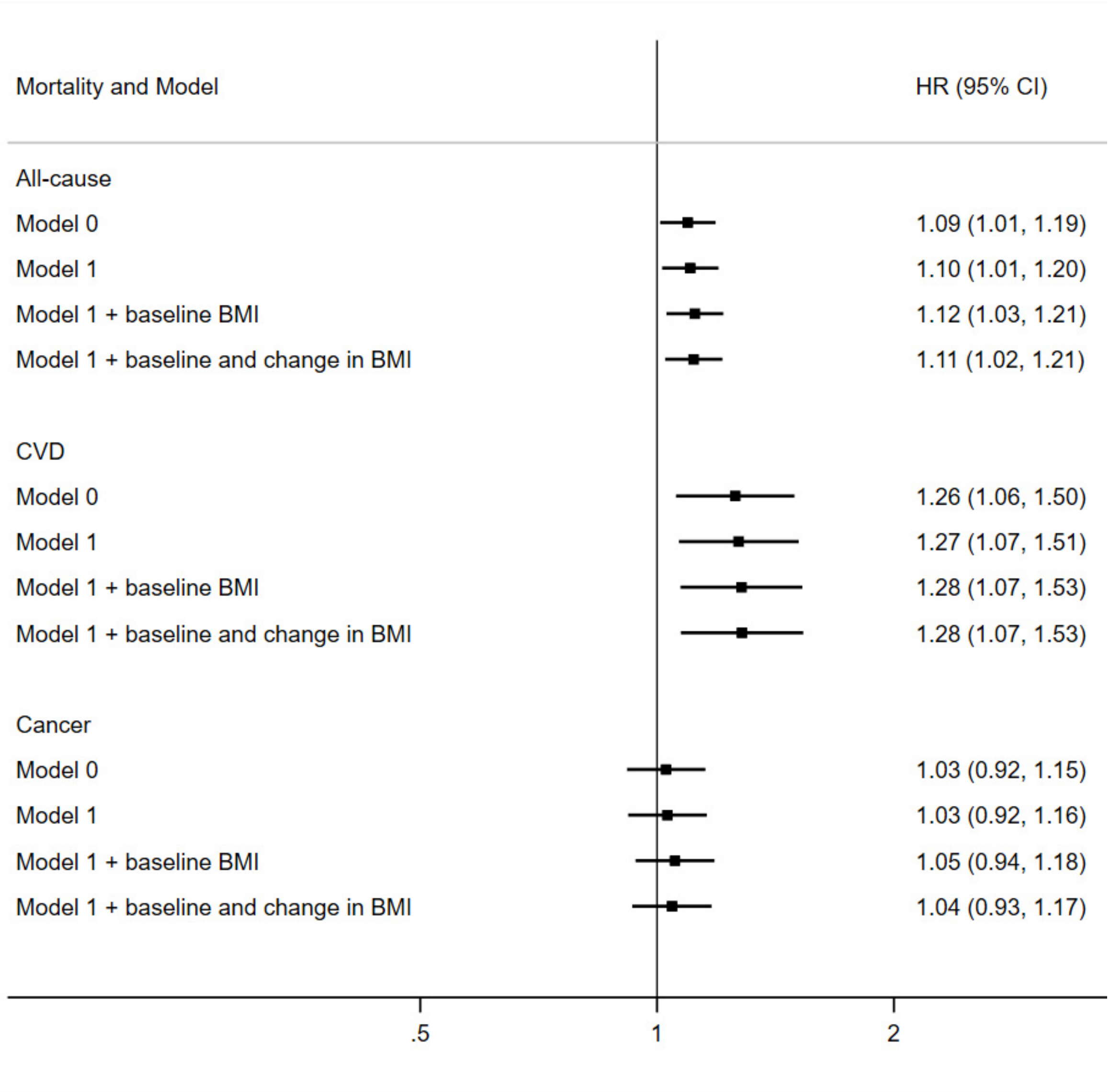

Model 0: stratified by sex, study centre and education level, and adjusted for baseline age, intervention group, alcohol intake, smoking status and cumulative average of Mediterranean diet score

Model 1: Model 0 + hypertension, hypertriglyceridemia, hypercholesterolemia, diabetes

Figure S5. Effect of adjustment for cardiovascular risk factors, baseline body mass index and change in body mass index on the estimates of associations with all-cause, cardiovascular and cancer mortality of change in energy intake, N=6,168, PREDIMED study

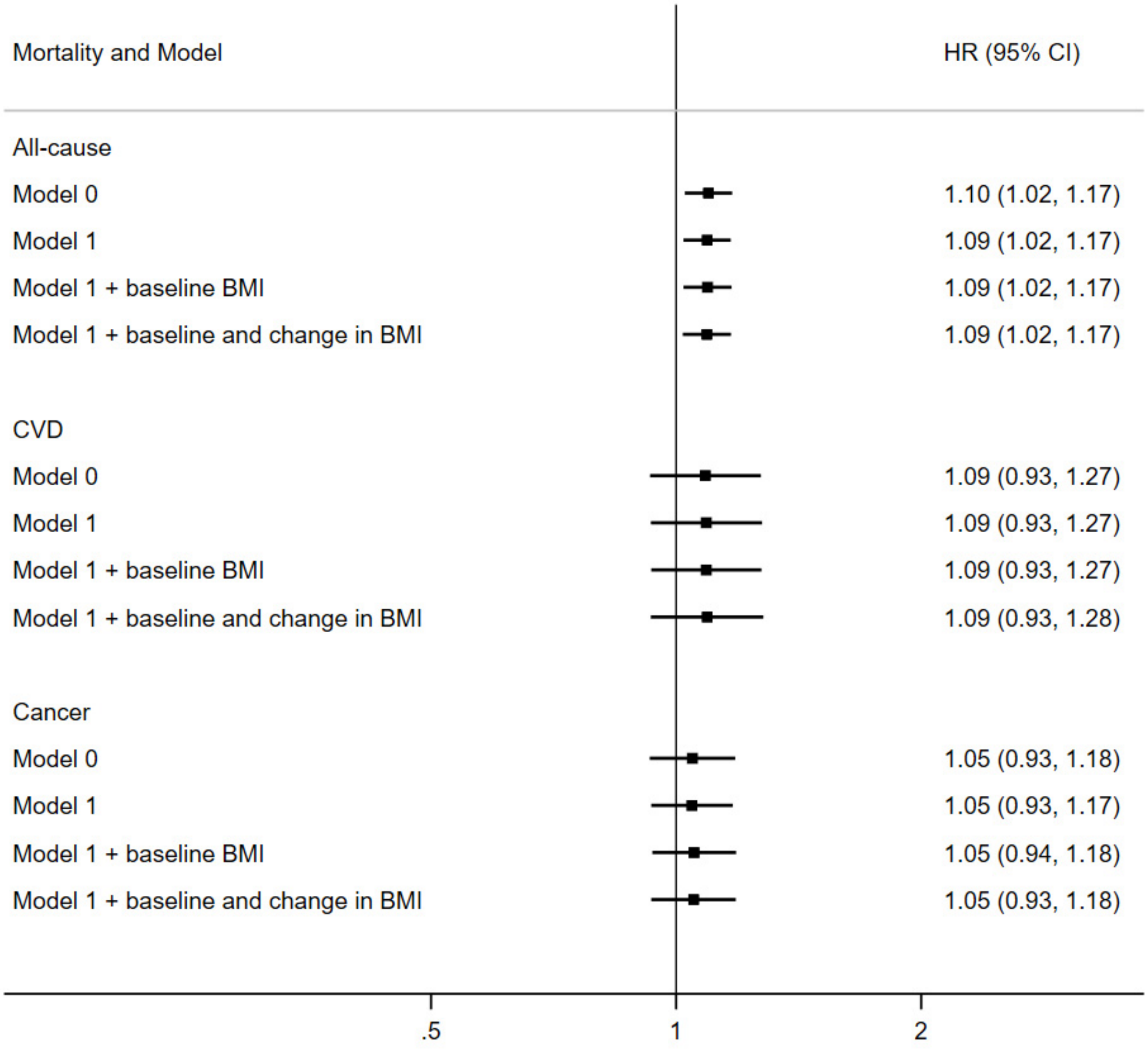

Model 0: stratified by sex, study centre and education level, and adjusted for baseline age, intervention group, alcohol intake, smoking status, physical activity and cumulative average of Mediterranean diet score

Model 1: Model 0 + hypertension, hypertriglyceridemia, hypercholesterolemia, diabetes

Full list of PREDIMED study collaborators

Hospital Clinic, Institut d'Investigacions Biomèdiques August Pi i Sunyer, Barcelona, Spain: R. Estruch, M. Serra, A. Pérez-Heras, C. Viñas, R. Casas, L. de Santamaría, S. Romero, E. Sacanella, G. Chiva, P. Valderas, S. Arranz, J.M. Baena, M. García, M. Oller, J. Amat, I. Duaso, Y. García, C. Iglesias, C. Simón, L. Quinzavos, L. Parra, M. Liroz, J. Benavent, J. Clos, I. Pla, M. Amorós, M.T. Bonet, M.T. Martin, M.S. Sánchez, J. Altirriba, E. Manzano, A. Altés, M. Cofán, C. Valls-Pedret, A. Sala-Vila, M. Doménech, R. Gilabert, and N. Bargalló.

University of Navarra, Primary Care Centres, Pamplona, Spain: M.Á. Martínez-González, A. Sánchez-Tainta, B. Sanjulián, E. Toledo, M. Bes-Rastrollo, A. Martí, C. Razquin, P. Buil-Cosiales, M. Serrano-Martínez, J. Díez-Espino, A. García-Arellano, I. Zazpe, F.J. Basterra-Gortari, E.H. Martínez-Lapiscina, A. Gea, M. Garcia-Lopez, J.M. Nuñez-Córdoba, N. Ortuño, N. Berrade, V. Extremera-Urabayen, C. Arroyo-Azpa, L García-Pérez, J. Villanueva-Tellería, F. Cortés-Ugalde, T. Sagredo-Arce, Mª D. García de la Noceda-Montoy, Mª D. Vigata-López, Mª T. Arceiz-Campo, A. Urtasun-Samper, Mª V. Gueto-Rubio, and B. Churio-Beraza.

University of Valencia, Valencia, Spain; Universitat Jaume I, Castellon, Spain; and Conselleria de Sanitat, Generalitat Valenciana: D. Corella, Guillem-Saiz P, P. Carrasco, C. Ortega-Azorín, E.M. Asensio, R. Osma, R. Barragán, F. Francés, M. Guillén M, J.I. González, C. Sáiz, O. Portolés, F.J. Giménez, O. Coltell (U. Jaume I), R. Fernández-Carrión, I. González-Monje, L. Quiles, V. Pascual, C. Riera, M.A. Pages, D. Godoy, A. Carratalá-Calvo, S. Sánchez-Navarro, and C. Valero-Barceló.

University Rovira i Virgili, Reus, Spain: J. Salas-Salvadó, M. Bulló, R. González, C. Molina, F. Márquez, N. Babio, M. Sorlí, J. García-Roselló, F. Martin, R. Tort, A. Isach,

B. Costa, J.J. Cabré, J. Fernández-Ballart, N. Ibarrola, C. Alegret, P. Martínez, S. Millán, J.L. Piñol, J. Basora, and J.M. Hernández.

Institut Hospital del Mar d'Investigacions Mèdiques, Barcelona, Spain: M. Fitó, M.I. Covas, O. Castañer, S. Tello, J. Vila, H. Schröder, R. De la Torre, D. Muñoz-Aguayo, N. Molina, E. Maestre, A. Rovira, R. Elosua, and M. Farré.

University Hospital of Alava, Vitoria, Spain: F. Arós, I. Salaverría, T. del Hierro, J. Algorta, S. Francisco, A. Alonso-Gómez, J. San-Vicente, E. Sanz, I. Felipe, A. Alonso-Gómez, and A. Loma-Osorio.

University of Málaga, Málaga, Spain: E. Gómez-Gracia, R. Benítez-Pont, M. Bianchi-Alba, J. Fernández-Crehuet Navajas, J. Wärnberg, R. Gómez-Huelgas, J. Martínez-González, V. Velasco-García, J. de Diego-Salas, A. Baca-Osorio, J. Gil-Zarzosa, J.J. Sánchez-Luque, and E. Vargas-López.

Institute of Health Sciences, University of Balearic Islands, and Hospital Son Espases, Palma de Mallorca, Spain: M. Fiol, M. García-Valdueza, M. Moñino, A. Proenza, R. Prieto, G. Frontera, M. Ginard, F. Fiol, A. Jover, and J. García.

Department of Family Medicine, Primary Care Division of Sevilla, Sevilla, Spain: J. Lapetra, M. Leal, E. Martínez, J.M. Santos, M. Ortega-Calvo, P. Román, F.J. García, P. Iglesias, Y. Corchado, E. Mayoral, and C. Lama.

University of Las Palmas de Gran Canaria, Las Palmas, Spain: L. Serra-Majem, J. Álvarez-Pérez, E. Díez-Benítez, I. Bautista-Castaño, I. Maldonado-Díaz, A. Sánchez-Villegas, F. Sarmiendo-de la Fe, C. Simón-García, I. Falcón-Sanabria, B. Macías-Gutiérrez, and A.J. Santana-Santana.

Hospital Universitario de Bellvitge, Hospitalet de Llobregat, Barcelona, Spain: X. Pintó, E. de la Cruz, A. Galera, Y. Soler, F. Trias, I. Sarasa, E. Padres, R. Figueras, X. Solanich, R. Pujol and E. Corbella.

Clinical End Point Committee: F. Arós (chair), M. Aldamiz, A. Alonso-Gómez, J. Berjón, L. Forga, J. Gállego, M. A. García-Layana, A. Larrauri, J. Portu, J. Timiraus, and M. Serrano-Martínez.
